# Replication of Vectored Herpesvirus of Turkey (HVT) in a Continuous, Microcarrier-Independent Suspension Cell Line from Muscovy Duck

**DOI:** 10.3390/vaccines13070714

**Published:** 2025-06-30

**Authors:** Karoline Mähl, Deborah Horn, Sirine Abidi, Benedikt B. Kaufer, Volker Sandig, Alexander Karlas, Ingo Jordan

**Affiliations:** 1 ProBioGen AG, Herbert-Bayer-Straße 8, 13086 Berlin, Germany; 2 Institut für Virologie, Freie Universität Berlin, Robert-von-Ostertag-Str. 7-13, 14163 Berlin, Germany; 3 Veterinary Centre for Resistance Research (TZR), Freie Universität Berlin, 14163 Berlin, Germany

**Keywords:** HVT, MDV, Marek’s disease vaccine, suspension cell culture

## Abstract

**Background/Objectives:** More than 33 billion chickens are industrially raised for meat and egg production globally and vaccinated against Marek’s disease virus (MDV). The antigenically related herpesvirus of turkey (HVT) is used as a live-attenuated vaccine, commonly provided as a recombinant vector to protect chickens against additional unrelated pathogens. Because HVT replicates in a strictly cell-associated fashion to low levels of infectious units, adherent primary chicken or duck embryo fibroblasts are infected, dislodged from the cultivation surface and distributed as cryocultures in liquid nitrogen to the site of application. Although viable cells are complex products, application of infected cells in ovo confers protection even in presence of maternal antibodies. **Methods/Results**: The aim of our study was to determine whether a continuous cell line in a scalable cultivation format can be used for production of HVT-based vaccines. The AGE1.CR cell line (from Muscovy duck) was found to be highly permissive in adherent cultures. Propagation in suspension, however, initially gave very low yields. The induction of cell-to-cell contacts in carrier-independent suspensions and a metabolic shock improved titers to levels suitable for vaccine production (>10^5^ infectious units/mL after infection with multiplicity of 0.001). **Conclusions:** Production of HVT is challenging to scale to large volumes and the reliance on embryonated eggs from biosecure facilities is complex. We demonstrate that a cell-associated HVT vector can be propagated in a carrier-independent suspension culture of AGE1.CR cells in chemically defined medium. The fed-batch production is independent of primary cells and animal-derived material and can be scaled to large volumes.

## 1. Introduction

Vaccination is a cost-effective prophylactic approach for the control of infectious diseases in animals to increase food security and reduce the economic burden from production losses. As poultry are often reared at very high population densities and for a short lifespan, the required vaccines must be produced in high volumes at very low cost. Ideally, a vaccine would provide multivalent protection via a single vector and be produced using a uniform manufacturing process [1].

The highly oncogenic Marek’s disease virus (MDV or *Gallid alphaherpesvirus 2*, GaHV-2, serotype 1) is an alphaherpesvirus of the genus *Mardivirus* that replicates in the nucleus of the infected cell and is transmitted predominantly via cell-to-cell contact [2,3,4]. MDV has increased in virulence since industrial poultry farming was introduced in the 1950s. The currently circulating strains induce fatal lymphomas with mortality rates of up to 100% in unvaccinated chicken and cause enormous economic losses of more than USD 1 billion per year in the poultry industry [5,6]. The closely related herpes virus of turkey (HVT or *Meleagrid alphaherpesvirus 1*, MeHV-1, serotype 3) naturally infects turkeys and is non-pathogenic in chickens. HVT is antigenically related to MDV and is commercially used as an avirulent live vaccine worldwide [7]. HVT can be used as a vaccine vector for the expression of antigens from other avian pathogens such as Newcastle disease virus (NDV), avian influenza A virus (AIV), infectious bronchitis virus (IBV), infectious bursal disease virus (IBDV) and infectious laryngotracheitis virus (ILTV). Examples for approved vaccines are Vectormune HVT-AIV (CEVA Santé Animale, Libourne, France, with EMA product number EMEA/V/C/006288), Innovax-ND-IBD (Merck Animal Health, Rahway, NJ, USA, EMEA/V/C/004422), Poulvac Procerta HVT-IBD-ND (Zoetis, Parsippany, NJ, USA, EMEA/V/C/006306) and the VAXXITEK^®^ platform (Boehringer Ingelheim Vetmedica GmbH, Ingelheim am Rhein, Germany, for example, EMEA/V/C/000065 for Vaxxitek HVT+IBD).

HVT is produced in adherent primary chicken or duck embryo fibroblasts and the distributed vaccines consist of viable infected cells stored as cryocultures in liquid nitrogen. Although such a format is logistically challenging, this cell-associated vaccine can be applied in ovo, is not inhibited by maternal antibodies and effective even in environments with high MDV prevalence. However, while vaccination prevents lymphoma and overt disease, it does not protect against virus replication and transmission [8]. A robust supply with vaccine remains essential for controlling Marek’s disease.

Primary avian cells for vaccine production are obtained from embryonated eggs that meet stringent biosecurity quality standards. The dependence on such a material is associated with high costs, complex logistics, lot variations and supply limitations [9]. Adherent cells that were previously reported to be permissive for *Mardivirus* originate from chickens such as DF-1, ESCDL-1 and JBJ-1, or quails such as QT-35 and QM-7 [10,11,12,13]. EB66 cells from the Pekin duck appear not to be permissive [14]. A disadvantage associated with gallinaceous cell lines is that chickens and quail carry endogenous retroviruses that might be mobilized, and form reverse transcriptase-containing particles during the production process. Some quail-origin cell lines may also carry latent genomes of quail-derived MDV that can be reactivated during the adaptation phase [15,16]. Muscovy ducks have a very low burden of endogenous retroviruses, and we did not detect any particle-associated reverse transcriptase activity in the cell lines derived from these animals.

Modern scalable and robust suspension production processes based on immortalized continuous cell lines would be highly desirable for high-volume vaccines [17,18,19,20]. To our knowledge, the propagation of the highly cell-associated HVT and other mardiviruses in immortal suspension cells and without animal-derived components has not yet been demonstrated. In this study, we quantified the susceptibility and permissivity of four continuous Muscovy duck cell lines for HVT and demonstrate a production process in carrier-independent suspension cultures.

## 2. Materials and Methods

### 2.1. Cell Lines

The derivation of adherent and suspension **a**denovirus **g**ene **E1**A/E1B-immortalized (AGE1) cell lines from the retina (AGE1.CR and AGE1.CR.pIX) and of adherent cell lines from somites (AGE1.CS and AGE1.CS.pIX) of Muscovy duck embryos (in the following CR, CS, CR.pIX and CS.pIX) was described previously [21]. The E1A and E1B genes are essential for maintenance of immortalization and can therefore serve as reference genes for copy number determination by ddPCR (Table 1). The CR.pIX and CS.pIX cell lines were further modified for stable expression of the minor capsid protein of human adenovirus serotype 5, the pIX protein [21,22]. UMNSAH/DF-1 cells were purchased from Cytio (Germany, catalog number 305016).

Adherent cells were maintained in Dulbecco’s minimal essential media (DMEM/F-12; Gibco/Thermo Fisher Scientific, Waltham, MA, USA), supplemented with 5% bovine serum (FBS) (Gibco/Thermo Fisher Scientific), incubated at 37 °C and 8% CO_2_ and split upon confluency with TrypLE Express Enzyme (Gibco/Thermo Fisher Scientific). CR and CR.pIX suspension cultures were maintained in chemically defined CD-U7 X02 medium (Xell/Sartorius AG, Bielefeld, Germany), supplemented with GlutaMAX^TM^ 100X (Gibco/Thermo Fisher Scientific) to a final concentration of 2 mM and LONG^®^R^3^ IGF-I (Repligen, Waltham, MA, USA) to 10 ng/mL and split to 0.8–1 × 10^6^ cells/mL every 3–4 days. Suspension cells were maintained in baffled shaker flasks at 37 °C, 8% CO_2_, 70% humidity and 180 rpm, with 5 cm amplitude (HT Multitron Cell shaking incubator, Infors AG, Bottmingen, Switzerland). Infection experiments with suspension cells were performed in flat-bottom flasks, as described below. Culture volumes were 20–60 mL in flasks with 125 mL total volume. Cell counts and percent viability were determined with the XcytoMatic^®^ 40 device (Chemometec, Allerod, Denmark).

No antibiotic/antimycotic agents were used for the cultivation of the cell lines to avoid masking low-level contaminations. The parental CR-derived suspension cell banks intended for vaccine production were exhaustively tested against avian, bovine and porcine adventitious agents according to GMP guidelines. Research cell banks were tested against contamination with mycoplasma (Minerva Analytik, Rangsdorf, Germany) and for sterility. Select CR-derived cell banks were furthermore tested by transcriptome analysis (PathoQuest, Paris, France) for adventitious agents and cytochrome c oxidase subunit 1 DNA barcoding (DSMZ, Braunschweig, Germany) for identity and purity. The duck cell lines were repeatedly shown by product-enhanced reverse transcriptase PCR to be free of reverse transcriptase activity associated with (endogenous) retroviral particles.

### 2.2. Infection of Adherent Cells

The HVT-EGFP reporter strain FC-126 has been described previously [23,24] and was used for all infections. Because HVT replicates in a strictly cell-associated format, infectious units were propagated and passaged by co-cultivation of uninfected with freshly revitalized infected cells. For the characterization of adherent cells, infection was performed either by application of 100 fluorescence-forming units (FFUs) onto 1-day old cell monolayers, or by co-seeding of 100 FFUs of infected with uninfected cells [25]. Seeding cell densities for pre-seeding experiments were either 1 × 10^5^ or 3 × 10^5^ cells/mL in 12 well-plates. Cell densities were doubled for co-seeding experiments.

Because CR cells were most permissive for HVT, these cells were used for seed virus generation, starting with a clone of HVT-EGFP that was plaque purified on CR cells over 7 passages. Viral stocks were obtained by infection of adherent CR cells by co-seeding to a multiplicity of infection (MOI) of 0.003 into T150 flasks. Medium was replaced with DMEM/F12 containing 1% FBS one day after co-seeding. Cells were detached with TrypLE Express at day 6 post-infection (dpi), cryopreserved in growth medium supplemented with 7.5% dimethyl sulfoxide and 25% FBS, and stored in liquid nitrogen. Infectious units were determined by focus-forming assays via co-cultivation of serial ten-fold dilutions with non-infected cells and evaluated at 5–6 dpi.

### 2.3. Infection of Suspension Cultures

To propagate HVT in suspension cells, 4 × 10^6^ CR or CR.pIX cells/mL in CD-U7 medium were inoculated to MOIs between 0.1 and 0.0001 with freshly revitalized seed virus preparation. A typical MOI in the literature appears to be 0.02 [13,25], the MOI range from 0.1 to 0.0001 was previously tested with adherent cells [26]. Aggregate formation was induced by addition of 1 volume of non-supplemented DMEM medium (Gibco/Thermo Fisher Scientific,) to support spread of the cell-associated infectious units [27]. Infected cultures were maintained in shaking incubators for up to 7 days, typically for 5 days. HVT-infected cell aggregates were dissociated by addition of EDTA to 2 mM out of a 500 mM stock solution (Invitrogen, Waltham, MA, USA) and incubation for 2–5 h at 37 °C. The resulting single-cell suspension was centrifuged for 5 min with 200× *g* and resuspended in DMEM/F12 containing 5% FBS prior to titration on adherent CR cells. Infected suspension cells were resuspended in CD-U7 medium containing 7.5% DMSO for cryoconservation.

### 2.4. DNA Extraction and Droplet Digital PCR (ddPCR) for Quantification of HVT Genome Copy Numbers

DNA was extracted either from the whole wells with adherent cells or 500 µL of suspension culture. The cell pellets were processed with the QIAamp DNA Blood Mini Kit (Qiagen, Hilden, Germany), according to the manufacturer’s instructions. DNA samples were further diluted in distilled water 100-fold to 10,000-fold, depending on expected template abundance. A set of specific primers (900 nM) and TaqMan probes (250 nM) (all oligonucleotides from TIB Molbiol Syntheselabor GmbH, Berlin, Germany) were used for ddPCR quantification using the QX200 Droplet Digital PCR system, reagents and QX Manager Software Standard Edition (v. 2.1.0.25) (Bio-Rad Laboratories Inc., Hercules, CA, USA), as previously described [26,28,29], with modifications for ddPCR using HEX/BHQ1 for the viral probes and YAK/BHQ1 for the cellular probe (Table 1).

### 2.5. Focus Quantification

Infected adherent cultures in microtiter plates were scanned with the NyONE Scientific (Synentec GmbH, Elmshorn, Germany) device. The number and size of fluorescent foci were determined with the NyONE Fluorescent Plaque Morphology (1F) (v. 0.9) module.

### 2.6. Software

Analysis and presentation of data was performed with Python 3.12.7 in the Jupyter Notebook version 7.2.2. Composite figures were created with Affinity Photo 2.6 (Serif Ltd., Nottingham, UK). The raw output of the NyONE Scientific plate scanner is monochromatic tiles. Contrasts were adjusted with the same settings within a series for improved visibility and a green color was overlayed for EGFP tiles for orientation.

## 3. Results

### 3.1. Co-Seeding (As Opposed to Overlay Infection) Appears to Improve Virus Transfer

**Figure 1:**
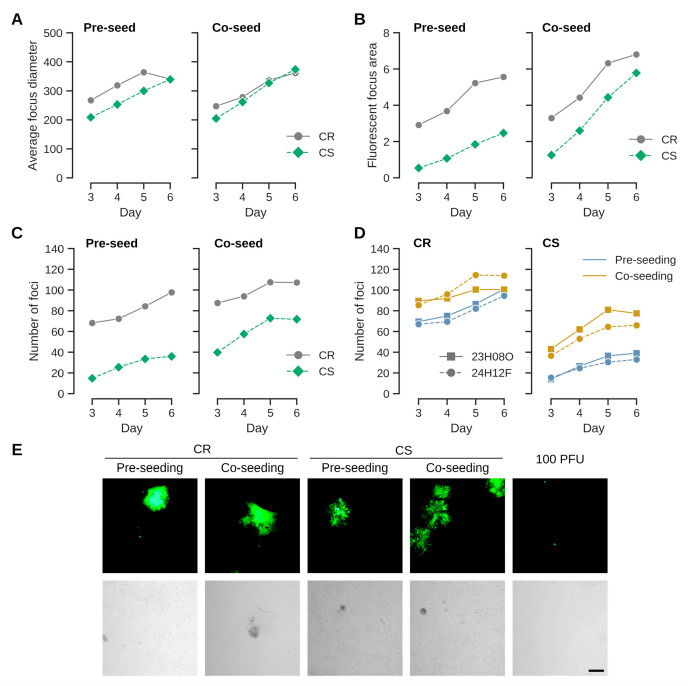
Uninfected cells were either **pre-seeded** one day prior to infection or **co-seeded** together with infected cells. The fluorescent foci were quantified by automated analysis with a plate reader. (**A**) Focus diameter (in µm) expand with a similar kinetic in both CR and CS cells independent of infection format. The usage of CR cells generally leads to larger (fluorescent) foci areas (in mm^2^) (**B**) and shows a higher number of foci (**C**) than CS cells. Although CR cell focus formation also improves by co-seeding, the effect is stronger for CS cells. Each curve is the mean of two independent experiments with seeding at higher cell density. (**D**) The effects appear not to be a chance event and were observed with different preparations of seed virus (23H08O and 24H12F; (**A**–**C**) were performed with the latter preparation) for both cell lines. (**E**) Example of foci obtained in CR and CS cell lines with either pre- or co-seeding. Foci generally appear to be more compact for CR cells than CS cells. Infection was performed with 100 FFU per well of a 12-well plate. Without uninfected cells, the seed did not expand into foci. The scale bar corresponds to 250 µm.

Mardiviruses are characterized by low productivities and strict association of infectious units with host cells [2,12]. They are therefore experimentally propagated by coculturing infected with uninfected cells [25]. The process can performed either by seeding infected cells onto an established monolayer of uninfected cells, or by mixing (co-seeding) infected and uninfected cells [18]. To test whether different infection protocols may be relevant for the continuous cell lines from Muscovy duck, we first compared effects on CR and CS cell lines that originate from different fetal tissues [21]. A benefit of co-seeding could be observed for both cell types but was stronger for CS cells (Figure 1). The effects were observed with different virus preparations in independent experiments. They were most pronounced when measuring the fluorescent area and number of foci, but less clear when comparing average focus diameters. This result suggests that once an infection is established, spread is as efficient among CR and CS cells (indicated by focus diameters and similar appearance of foci independent of seed protocol (Figure 1E)). Although the susceptibility of CS cells for infection (indicated by the number of foci, and by correlation, the area of fluorescence) appears to increase with co-seeding, the lower susceptibilities of CS cells compared to CR cells remained and were statistically significant in a one-way ANOVA, with *p*-values < 0.005, independent of format (pre- versus co-seeding), virus preparation or cell density for the endpoint at day 6. The differences in pre- compared to co-seeding for the number of foci were also statistically significant for CS cells with *p*-values of 0.0002 (high cell densities) and 0.0033 (low cell densities). The differences for pre- compared to co-seeding were not statistically significant for CR cells (*p*-values 0.3948 and 0.1157).

Seeding the same amount of the virus preparations as was used for infection (100 FFUs per well of a 12-well plate) without pre- or co-seeding of uninfected cells did not result in cell foci (Figure 1E).

### 3.2. HVT Infects and Replicates in Adherent Cairina Moschata Cell Lines from Different Lineages

**Figure 2:**
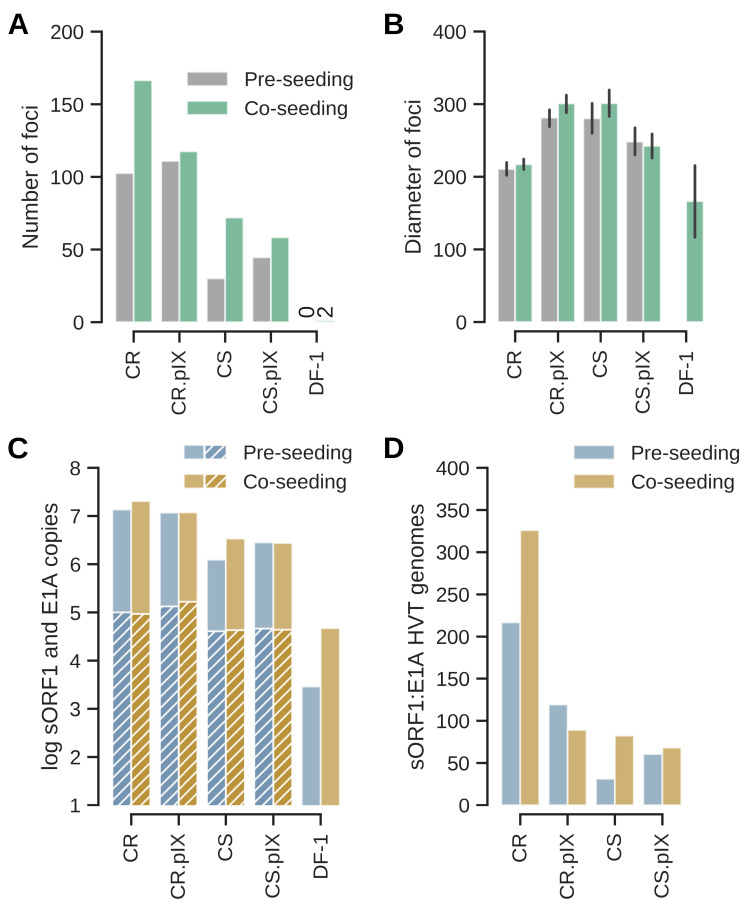
Endpoint observations at day 7 after infection by pre- or co-seeding with 100 FFU per well of a 12-well plate. (**A**) Co-seeding leads to higher number of foci also for the CR- and CS-derived cell lines that express the pIX protein. DF-1 cells are barely susceptible to HVT-GFP and only 2 foci were obtained by co-seeding. (**B**) Focus diameters (in µm) show similar expansion at day 7 but are surprisingly low for CR cells. This effect may possibly be caused by loss of some expanded foci to cytopathic effect because of the long incubation until day 7 (see also Figure 3). The error bars show standard deviation based on number of foci detected per respective well shown in (**A**). (**C**) Genome copy numbers for HVT are consistent with the observed number of foci. Because the DF-1 cell line does not encode the E1A gene that was used to immortalize the CR cell line [21], absolute numbers are given here for HVT and (where appropriate, hatched columns) for E1A. (**D**) Genome copy numbers normalized to the cellular E1A reference. Absolute copy numbers are shown on a log scale, all other values on a linear scale. Results shown here are the averages of two independent experiments.

CR and CS cells were derived from different tissues: the CR from the retina and the CS cells from embryonal somites [21]. That co-seeding is generally associated with a higher efficiency of infection appears not to be a clonal effect and was confirmed for CR and CS cell lines that stably express a minor structural protein of human adenovirus serotype 2, the pIX protein (Figure 2). This pleotropic protein appears to be involved in reorganizing host cell nuclear domains [22] and heat shock responses [21], and we suspected previously that the presence of pIX may interfere to a stronger extent with replication of HVT but less with CVI988/Rispens as this strain replicated in both cell lines [26]. CVI988/Rispens is an avirulent, cell-culture adapted clone of an isolate of serotype 1 MDV that appears to establish a longer persistence in animals compared to serotype 3 HVT [30,31,32]. The cytopathic effect caused by HVT was very low in the first passages so we may have missed the infection in the CR.pIX cells at that time and did not passage the infected cells appropriately. Here, assisted by the GFP-fluorescence, we found that all Muscovy duck cell lines can be productively infected with HVT, and that co-seeding tends to yield large number of foci (Figure 2A). The DF-1 cell line was barely susceptible or permissive in our experiments. The results obtained for fluorescent foci were confirmed by ddPCR against the viral sORF1 gene and for the designed duck cell lines against E1A as a cellular reference (Figure 2C).

**Figure 3:**
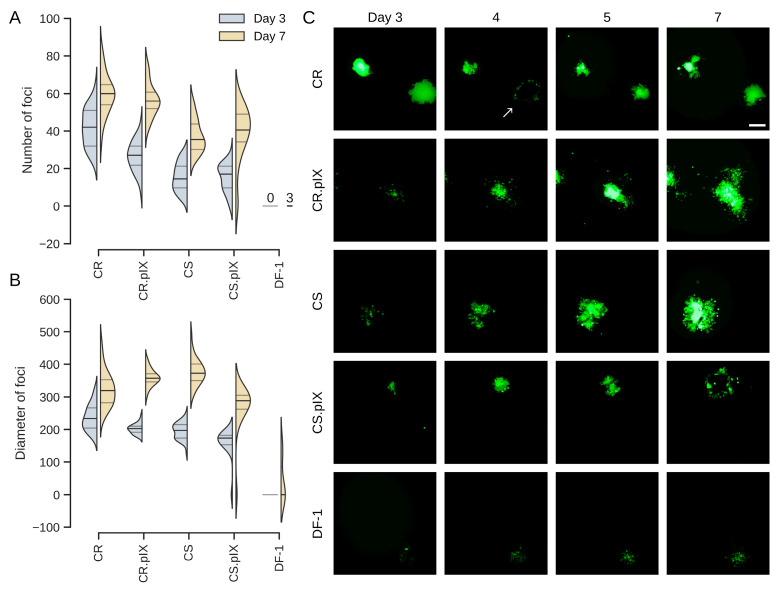
Number of foci (**A**) and size of foci in µm (**B**) compared between day 3 and day 7 after infection. The bold line in the violin plot depicts the median value, the thin lines the upper and lower quartiles. Appearance and development of foci from day 3 to day 7 (**C**). The arrow at day 4 in the row with CR cells points to a focus that developed into a true plaque where cells in the center dislodge. Such plaques are usually regrown with fresh cells that are also infected. The scale bar corresponds to 250 µm.

Having confirmed that co-seeding is the more efficient infection protocol, we performed plaque size assays to quantify permissivities (Figure 3). Uninfected CR, CR.pIX, CS, CS.pIX and DF-1 cells, respectively, were seeded to 0.5 × 10^6^ cells per well into two rows each of a 24-well plate together with 50 FFU each of freshly revitalized seed virus from cryopreserved stocks (12 wells total per cell line). Medium was replaced on the following day and on days 4 and 7 with medium containing only 1% FBS. The infected cultures were scanned daily with the NyONE plate reader. As shown in Figure 3C (and similar to Figure 1E), focus morphologies were again slightly different between CR and CS cells. The foci were more compact for CR and more diffuse, especially at the edges, for CS cells.

A confounding factor in the determination of focus counts is that, on occasion, cells in the center of a focus detach, leaving a plaque with a faint fluorescent rim. Infected cells grow back into this area so that the fluorescent focus is restored. However, depending on the timing of the assay, the advanced foci can be difficult to register by human operators or the NyONE plate reader. Although we started the experiments with a plaque-purified virus, we furthermore underestimate the number of foci by quantifying fluorescence because not all foci expressed EGFP (see also Figure 6C). With these limitations in mind, CR cells were again more susceptible, followed by CR.pIX and CS cells. Permissivity (focus diameters) was similar for the Muscovy duck cell lines. Only three foci were obtained with DF-1 cells.

### 3.3. HVT Can Be Propagated in Microcarrier-Independent Suspension Cells

Establishment in scalable suspension cultures is a key factor for the development of a consistent and affordable supply of high-volume vaccines. Because HVT is a cell-associated herpesvirus, the process in single-cell suspension must be designed in such a way that the frequency and duration of cell-to-cell contacts are sufficient for propagation. A scalable process has been described for the production of CVI988/Rispens (not HVT) and is based on adherent DF-1 cells [13]. However, bovine serum is required in the medium of the infected DF-1 cells that are furthermore cultivated on microcarriers. Dependence on bovine serum complicates risk-assessments and increases costs, and microcarriers can interfere with subsequent downstream steps such as harvest and cryoconservation. Here, we tested if carrier-independent suspension cultures in chemically defined medium can be used for the amplification of HVT.

**Figure 4:**
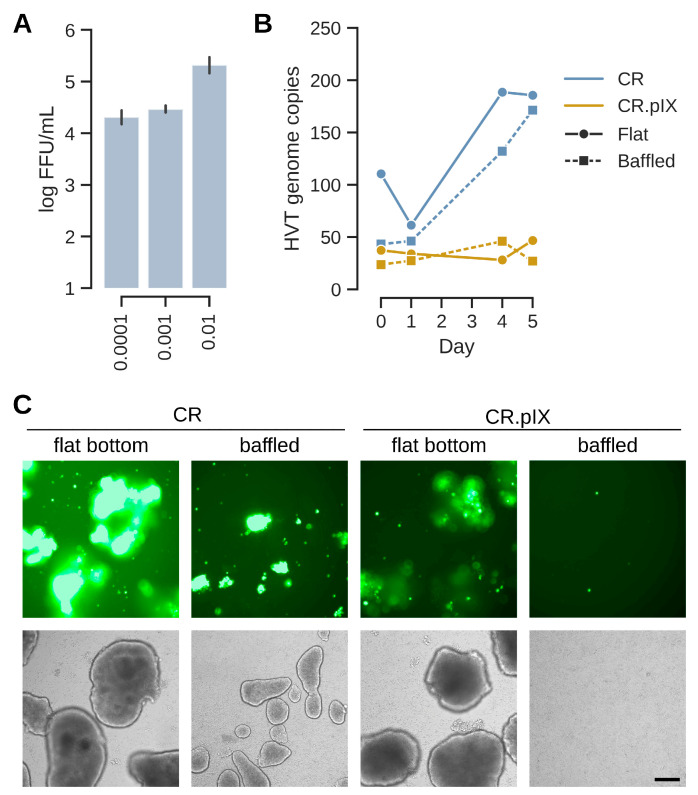
(**A**) Suspension CR cells in a non-baffled 125 mL-shaker flask were infected with MOIs from of 0.0001 to 0.01 and infectious titers were determined for samples at day 7 post-infection. Optimization of the suspension process was continued with the central MOI of 0.001. In (**B**) and (**C**), CR and CR.pIX infection was compared in baffled and flat-bottom shaker flasks. (**B**) Genome copy numbers (sORF1 copies normalized to E1A copies) increased during the infection in CR cells but remained essentially constant at the input level for CR.pIX. (**C**) While EGFP signals were strong in aggregates in CR cells infected in flat-bottom shaker flasks, aggregates were fewer and smaller in baffled flasks and EGFP signals less pronounced. CR.pIX cells did not perform well even in non-baffled shaker flasks. The scale bar corresponds to 250 µm.

Only CR and CR.pIX cells were included in this phase, because CS cells cannot be adapted to anchorage independent growth [21]. In a first screening experiment, suspension CR cells were infected with MOIs of 0.01, 0.001 and 0.0001, respectively, using a fed-batch process that was demonstrated earlier to be highly efficient for production of poxviruses [27]. This process relies on induction of cell aggregates in a biphasic process with chemically defined components. The suspended cell aggregates form without microcarriers and allow the spread of cell-associated infectious units. For HVT, the yields were 2.2 × 10^4^, 3.0 × 10^4^ and 2.3 × 10^5^ FFU/mL for MOIs of 0.0001, 0.001 and 0.01, respectively (Figure 4A). With 2 × 10^6^ cells/mL at the time of infection the above MOIs translating into input-to-yield ratios of 111-, 15- and 12-fold. This confirms that HVT replicates in these cells also at very low MOIs but at this stage of the development, we decided to continue optimization with the central MOI of 0.001 as a starting point.

In bioreactors, the size of aggregates can be controlled with the type of impeller and stirring speed. Here, to first test the impact of aggregate size, formation was investigated in flat bottom and baffled shake flasks (Figure 4B,C). Samples were taken directly after infection and at days 1, 4 and 5 for genome copy number determination. The E1A gene that was used to immortalize the CR cell line [21] served as an internal reference to control for cell expansion during the infection and stochastic effects when sampling in a suspension that may be heterogeneous due to the presence of aggregates. Aggregates formed in baffled shake flasks were smaller and the spread of GFP signal was not as pronounced compared to those in flat-bottom flasks (Figure 4C). The differences between CR and CR.pIX cells were stronger in suspension compared to adherent format with CR cells appearing to be more permissive. This observation was reflected in higher genome copy numbers for CR cells in a non-baffled flask, and for CR in both flask formats when compared to CR.pIX (Figure 4B). The mean aggregate dimensions in flat-bottom flasks were 518 ± 164 µm × 739 ± 177 µm (width × length).

### 3.4. Metabolic Stressor Improves Titers

**Figure 5:**
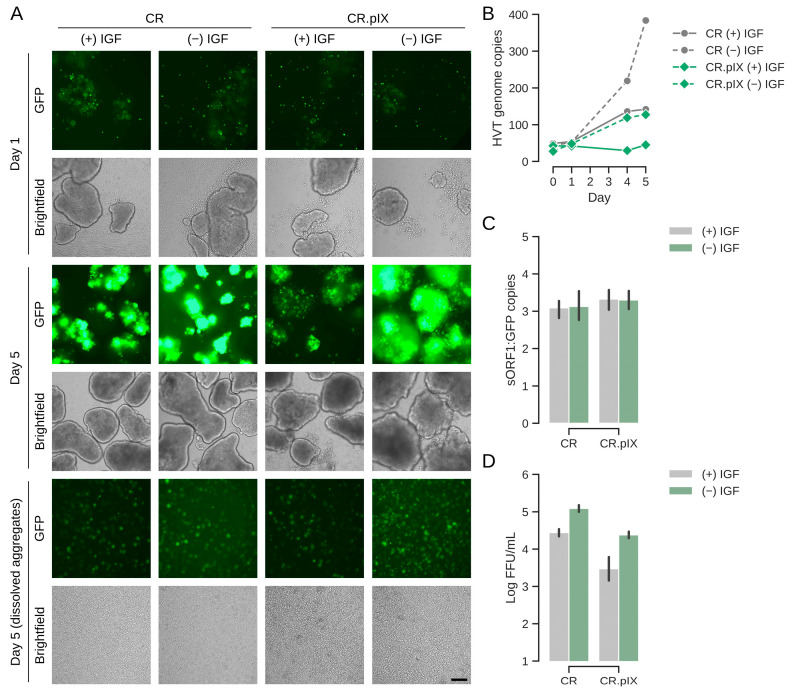
Sudden depletion of IGF improves titers significantly. (**A**) EGFP signal increased especially strongly in aggregates of CR and CR.pIX cells in the shock-depleted cultures. The first panel shows cultures 1 day after infection, the center panel at day 5 and the bottom panel the harvest also at day 5 after dissociation of aggregates. The scale bar corresponds to 250 µm. (**B**) Viral genome copy numbers (sORF1 copies normalized to E1A) increased to highest levels in shock-depleted CR cultures, followed by normal CR and shock-depleted CR.pIX cultures. (**C**) Viral genome copy numbers were determined by ddPCR against the EGFP transgene and the viral sORF1 gene. Ratios of sORF1 to EGFP were approximately 3 throughout the cultures in both cell lines. This ratio is consistent with non-fluorescent to fluorescent foci in titrations. (**D**) Infectious titers in FFU/mL obtained with the dissociated aggregates. The infectious titers may be underestimated by a factor of 3.

Infectious titers well above 10^4^ FFU/mL and normalized copy numbers in the range of 200 per cell are good for a virus with the low productive infectious cycle of HVT but still need to be improved for achieving a commercially viable process. In a natural infection, mardiviruses (including HVT) can integrate into the genome of infected cells to establish latency [33], which aids dissemination within the host and may also contribute to the long-lasting protection of CVI988/Rispens- and HVT-based vaccines [4,24,34,35]. Only 1–10% (and occasionally none) of cultured lymphoblastoid cell lines that contain latent mardiviruses are in the lytic phase of infection and the mechanisms of mobilization are not fully understood [36]. Integration of MDV into the host genome appears to be a frequent and rapid event [37]. Although such observations are not fully transferrable to our experiments that start with an acute infection in a continuous, non-lymphoid cell line, we hypothesized that infectious titers possibly can be improved if lytic replication, or reactivation in case of integration, can be increased by performing and maintaining the infection under metabolic or proteotoxic stress. After different attempts to improve lytic replication with ethanol and temperature shock, or metabolic inducers such as sodium butyrate, agmatine or the phosphatidylinositol 3-kinase (PI3K)/Akt pathway activator YS-49 [38] failed, we tested removal of LONG-R3 IGF-I. This growth factor has pleotropic effects that range from PI3K activation to inhibition of apoptosis [39]. It is the only proteinaceous component of the chemically defined suspension medium and although CR and CR.pIX cells can be adapted to proliferation in depleted medium, IGF-I is essential for routine maintenance. As shown in Figure 5, a sudden and complete IGF I-depletion at the time of infection appeared to be highly beneficial. After dissociation of cell aggregates, the infectious titers in the depleted medium were 1.3 × 10^5^ FFU/mL. The genome copy numbers (normalized against the cellular E1A gene) increased to 400. Consistent with the observation of non-fluorescent foci in the titration plates, the determined genome copy numbers of the vector-encoded EGFP transgene and the viral sORF1 gene gave a ratio of approximately 1:3. Counting non-fluorescent and fluorescent foci in selected wells by eye gave similar ratios. These observations suggest that some of the viruses may have lost the mini-F replicon core (containing the pTK-eGFP reporter cassette), which was used to generate the recombinant construct [24]. Only FFU were determined because non-fluorescent foci are not reliably detected because of the weak cytopathic effect, most likely with the risk of underestimating titers three-fold.

The effects were strong for both the CR and CR.pIX cell line; however, with higher titers and genome copy numbers in the parental CR cell line. The visible increase in GFP signal from day 1 to day 5 in the induced aggregates is consistent with the quantified parameters for virus replication (Figure 5A). Lower gene copy numbers for CR.pIX than CR in comparable settings mirror the results obtained in the adherent format.

**Figure 6:**
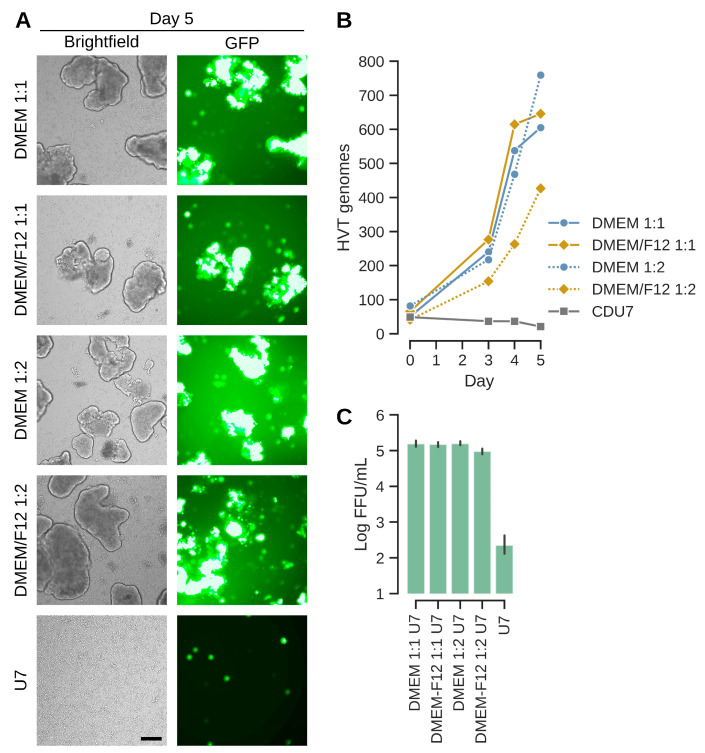
The suspension process tolerates differences in production media composition and ratios. (**A**) EGFP signal distribution and aggregate appearances at the day of harvest prior to dissociation. Ratios (such as 1:1) refer to amount production medium added relative to the suspension medium CD-U7. The bottom panel depicts the experiment where no production medium (only 1 volume CD-U7) was added. The scale bar corresponds to 250 µm. (**B**) Genome copy numbers increase, especially starting at day 3. Values were best for DMEM as production medium or if DMEM/F12 is given at least at 1:1. (**C**) Infectious titers at day 5 were above 10^5^ FFU/mL for the three cultures that exhibited strong genome copy number increases. Aggregate induction is important as shown by infectious titers in the CD-U7 only culture that correspond to less than input virus (infection with MOI 0.001 × 2 × 10^6^ cells/mL at the time of infection equals 2 × 10^3^ FFU/mL).

The above results were confirmed with a variation in the aggregate-inducing production medium in the fed batch process (Figure 6). As the previous experiment suggested better results with larger aggregates, we also reduced the ratio of production media to test whether less densely packed aggregates might confer an advantage for replication. The infections were performed with MOIs of 0.001 and IGF-depletion in normal (non-baffled) shaker flasks. As shown in Figure 6, titers were above 10^5^ FFU/mL in all cases where virus production medium was added. Consistent with the previous experiment with baffled flasks, the formation of aggregates is important for replication. If no aggregates are induced, then titers are below the level of input virus after 5 days of incubation. E1A-normalized genome copy numbers were 605–760 for the three best experiments and mirror the infectious titers. Such numbers correspond to 10-fold higher genome copy numbers compared to chicken feather follicle cells (2 × 10^8^ genomes per million cells in CVI988-vaccinated animals [40]; 605 × 2 million cells, 1.2 × 10^9^ sORF1 copies in CR cells). Yields were slightly lower in presence of DMEM/F12 at 1:2 ratio, but overall, the process appears to be resilient against variations in ratios or composition of the production medium. The GFP signal distribution in the aggregates is consistent with a good spread of infectious units. However, not all aggregates and not all regions within aggregates show green fluorescence. This observation potentially points to a limitation of the current process.

## 4. Discussion

The continuous CR and CS duck cell lines can be productively infected with HVT. Viral replication was best in retina-derived CR cells, followed by CR.pIX, and then by the embryonal somite-derived CS and CS.pIX. We also demonstrate that HVT can be cultivated in carrier-independent suspension cultures, again with higher yields in CR than CR.pIX cells. The cell lines were previously described as a potential replacement for primary cells for the cultivation of different avian pathogens, including an HVT-vectored NDV vaccine [26,41]. CR cells were permissive for HVT and strain CVI988/Rispens, while CR.pIX cells appeared to be permissive only for strain Rispens and surprisingly did not allow propagation of HVT [26]. The discrepancy to the current result may be due to the lower amount of inoculum in the earlier experiments, differences in strain passage history, and/or improved use of co-cultivation techniques. The earlier study was additionally performed by serial passaging of the infected cells, which may have promoted cycles of viral genomic integration and reactivation [24]. Based on the current results, it would be interesting to test whether chronic infections can be established in suspension cultures and whether the number of transferable infectious units is influenced by levels of integrated viral genomes.

Suspension cultures are more flexible in an industrial context. With similar footprints, manufacturing output can be adjusted via the working volume of bioreactor vessels and process intensification such as high cell density seeding and perfusion [42]. Challenges associated with adherent cells, and especially with primary cells, are the dependence on bovine serum and fixed bed or microcarriers for cultivation. While microcarriers may facilitate process steps such as exchange of medium, they increase complexity if the infected cells need to be harvested and the product is to be used for animals intended for human consumption [43]. A continuous influx of primary cells and bovine serum into a process introduces costs, is a potential source for contamination and increases the risk for batch consistency and supply security [42].

Mardivirus replication and morphogenesis appears to be inefficient; intracellular yields are low and free enveloped extracellular virions cannot be detected on infected chicken embryonic skin cell monolayers [12,44]. CVI988/Rispens was previously propagated in DF-1 suspension cultures on microcarriers with yields of 6.5 × 10^5^ infectious units (PFU/mL) after infection with an MOI of 0.02 [13]. The harvested viruses were tested in vivo and conferred immunological protection to SPF chickens.

In our study, we demonstrate that the higher-attenuated serotype 3 *Meleagrid alphaherpesvirus 1* (HVT) can be produced in anchorage-independent suspension cell cultures in chemically defined medium, with yields also above 10^5^ infectious units (FFU/mL). To facilitate direct cell-to-cell transmission of infectious units, we induced cell aggregates in the suspension cultures using a process developed for the scalable production of poxviruses [27]. The cell aggregates are formed without the aid of microcarriers and are maintained for the duration of the process in a stirred tank or shaken bioreactors. The cells are lysed prior to downstream processing in poxvirus purification processes. For the production of HVT-based vaccines, the aggregates are dissolved by addition of EDTA prior to cryoconservation of viable cells. Another difference is in the metabolic shock. Recombinant IGF-I is an expensive cell culture media component, so removal of this substance is advantageous. However, the timing for IGF-I depletion during the seed train towards the final production volume needs to be determined for an industrial process.

Additional limitations of our study need to be addressed in future experiments. First, we had to propagate virus with cell-contained infectious units. Cell-free HVT is not easily obtained [44] and less interesting for actual application because it appears to be not as protective as a cell-associated vaccine, probably due to interfering maternally derived antibodies [45,46]. Because seed virus was prepared in adherent CR cells, infected mixed cultures cannot be ruled out, albeit with a very low amount of input virus (MOI 0.01 or lower). Subsequent studies should include approved vaccine preparations and focus on the generation of a low-passage seed virus in suspension. Second, although plaque titration assays gave meaningful results in our study, an indication that transmission of virus from suspension to adherent cells was successful, animal experiments are required to demonstrate protective effects of the preferred in ovo vaccination with viable, virus carrying cells. Challenge experiments with a vaccine obtained from a microcarrier-independent suspension culture are also necessary to determine the potential impact of pre-existing maternal antibodies on vaccine potency, and to investigate options for multivalent preparations [45,46,47].

## 5. Conclusions

CR and CR.pIX cell lines can be productively infected with an EGFP-expressing HVT in carrier-independent suspension in chemically defined media. The resulting genome copy numbers and virus yields are similar to those reported for conventional permissive adherent cells. HVT is an important vector vaccine platform that can be functionalized for protection against a diverse set of poultry pathogens. The vector backbone itself contributes to the control of Marek’s disease, a globally prevalent devastating disease for backyard and industrial poultry farming. Production of the cell-associated vaccine is challenging, and a process based on continuous suspension cell lines may help to overcome the scalability limitations imposed by the current manufacturing processes based on embryonated eggs.

## Figures and Tables

**Table 1:** Primers and probes used in this study for droplet digital PCR (ddPCR).

Target	5′→3′
sORF-1 forward	GGCAGACACCGCGTTGTAT
sORF-1 reverse	TGTCCACGCTCGAGACTATCC
sORF-1 probe	HEX-AACCCGGGCTTGTGGACGTCTTC-BHQ1
GFP forward	GCACAAGCTGGAGTACAACTA
GFP reverse	TGTTGTGGCGGATCTTGAA
GFP probe	HEX-CAAGCAGAAGAACGGCATCAAGGC-BHQ1
E1A forward	TGACTCCGGTCCTTCTAACACA
E1A reverse	TCACGGCAACTGGTTTAATGG
E1A probe	YAK-CCCGGTGGTCCCGCTGTGC-BHQ1

## Data Availability

Data are available upon justified request.
